# Career Odysseys panel - Chrencik

**DOI:** 10.1063/4.0000859

**Published:** 2025-10-27

**Authors:** Jill Chrencik

**Affiliations:** Merck, South San Francisco, CA, USA

## Abstract

I am currently serving as a Senior Principal Scientist in the Discovery Chemistry organization at Merck, specifically with a role in the Protein and Structural Chemistry department. In my role, I focus on advancing medicinal chemistry design strategies through structure-based drug design. Our team utilizes a variety of structural techniques, including X-ray crystallography, cryo-electron microscopy (cryo-EM), hydrogen-deuterium exchange (HDX), bio-nuclear magnetic resonance (bio-NMR), and small-angle X-ray scattering (SAXS) that enable us to uncover intricate structural details of essential protein therapeutic targets.

I earned my Bachelor of Science in Chemistry from Syracuse University and completed my Ph.D. at the University of North Carolina at Chapel Hill. Following this, I pursued a postdoctoral fellowship at The Scripps Research Institute. Over the past seven years at Merck, I have had the privilege of leading a small structural biology team at our research site in South San Francisco. Prior to my tenure at Merck, I gained valuable experience at Pfizer, where I worked extensively on G protein-coupled receptors (GPCRs) and kinases. Additionally, I contributed to a startup company called Receptos, which specialized in GPCR research.

Beyond my scientific endeavors, I am actively involved in the South San Francisco Women’s Network and co-lead an internal initiative called #IamRemarkable (from Rmrkblty). This program is designed to support and empower women and individuals from underrepresented groups to advocate for themselves. I am deeply passionate about fostering increased representation and equity for women in science, and I strive to uplift my colleagues at Merck to create a more inclusive community.

